# Effects of vitamin C on production performance, rumen fermentation, and blood antioxidant indices in heat-stressed Holstein dairy cows

**DOI:** 10.3389/fvets.2026.1696533

**Published:** 2026-04-22

**Authors:** Hongsheng Du, Mengen Zhang, Guoqiang Ma, Yingli Li, Ke Zhou, Guodong Li, Fei Xie, Rubing Lan, Dian Wang

**Affiliations:** 1 Youran Dairy Co., Ltd., Hohhot, China; 2 College of Animal Science, Inner Mongolia Agricultural University, Hohhot, China; 3 Dairy Cow Breeding and Husbandry Technology Research Center, National Dairy Technology Innovation Center, Hohhot, China

**Keywords:** ascorbic acid, Holstein cows, milk performance, oxidative stress, thermal stress

## Abstract

Heat stress exerts severe detrimental effects on lactation performance, rumen health, and antioxidant status in dairy cows, inflicting substantial economic losses on the global dairy industry. This study investigated the effects of dietary Vitamin C-2-phosphate supplementation on lactation performance, rumen fermentation, and serum antioxidant parameters in heat-stressed Holstein cows. Thirty multiparous Holstein cows (659.6 ± 18.4 kg body weight, 2.83 ± 0.65 parity, 93.3 ± 9.3 days in milk, 44 ± 4.3 kg/d milk yield) were randomly allocated to three groups (*n* = 10) using a randomized complete block design: control (CON, basal diet), low-dose Vitamin C-2-phosphate (LVC, basal diet + 20 g/head/d Vitamin C-2-phosphate), and high- dose Vitamin C-2-phosphate (HVC, basal diet + 40 g/head/d Vitamin C-2-phosphate). The 42-day trial consisted of a 7-day adaptation period and a 35-day experimental period, with an average temperature-humidity index (THI) of 77.86. Results showed that Vitamin C-2-phosphate supplementation significantly increased ruminal total volatile fatty acid (TVFA) and acetate concentrations, energy-corrected milk (ECM) yield, milk protein concentration, and milk protein yield (*p* < 0.05), while decreasing ruminal ammonia nitrogen (NH_3_-N) concentration and milk somatic cell count (SCC) (*p* < 0.05). Additionally, Vitamin C-2-phosphate supplementation reduced serum malondialdehyde (MDA) and heat shock protein 70 (HSP70) levels and enhanced total antioxidant capacity (T- AOC) (*p* < 0.05). In conclusion, dietary Vitamin C-2-phosphate effectively improves rumen fermentation, alleviates oxidative stress, enhances lactation performance, and promotes mammary gland health in heat-stressed Holstein cows. The 40 g/head/d dosage yielded the most favorable effects among the tested levels. Future studies should determine the optimal Vitamin C-2-phosphate inclusion levels under different heat stress intensities and physiological stages of production, and explore its synergistic effects with other functional additives.

## 1 Introduction

In recent years, the global population growth and rapid industrialization have driven massive greenhouse gas emissions, exacerbating global warming. Holstein cows, being thermosensitive and poorly tolerant to heat, face significant challenges in dairy farming under rising temperatures (1). Heat stress—defined as the physiological and behavioral responses of dairy cows to high ambient temperatures—manifests as increased respiration rates, hyperthermia, and reduced feed intake (2). These adaptations facilitate heat dissipation and lower metabolic heat production, thereby maintaining homeostasis (3). However, the consequent energy deficit from increased maintenance requirements and decreased intake has led to declines in productive performance and substantial economic losses. Notably, reduced feed intake alone explains only 35–50% of this productivity drop (4). Under heat stress, studies report elevated reactive oxygen species (ROS) and malondialdehyde (MDA) levels alongside diminished superoxide dismutase (SOD), catalase (CAT), and total antioxidant capacity (T-AOC), disrupting redox balance (5). ROS accumulation disrupts cellular lipid and protein metabolism, triggering intestinal epithelial and mammary gland cell damage/apoptosis, which critically contributes to reduced milk yield per cow (6). Oxidative stress further modifies the structural integrity of milk bioactive molecules (e.g., *β*-lactoglobulin), compromising nutritional value and sensory properties (7). Addressing heat stress prevention and mitigation strategies thus represents an urgent priority for sustainable dairy farming in temperate regions.

The temperature-humidity index (THI) serves as a standardized metric for assessing heat stress severity, with dairy cows experiencing thermal discomfort at THI values exceeding 68 (8). While physical interventions—including shading, forced ventilation, and sprinkler cooling—are widely implemented to mitigate ambient temperatures in livestock systems, their efficacy remains suboptimal. Notably, sprinkler systems may exacerbate hoof disorders and mastitis incidence (9). Given these limitations in physically alleviating heat stress and its consequential oxidative burden, nutritional strategies targeting physiological homeostasis assume critical importance. For instance, dietary supplementation of *Litsea cubeba* essential oil has been shown to enhance antioxidant activity and immunity in fattening pigs, subsequently improving production metrics (10). Similarly, poultry studies conducted by Liu et al. (11) demonstrated that source-differentiated plant tannins modulated broiler gut microbiota, antioxidant status, and immune responses, reinforcing the therapeutic potential of botanical antioxidants. Despite these promising outcomes, the commercial adoption of such plant extracts faces constraints due to intricate manufacturing protocols and associated economic barriers. Comparatively, vitamin C emerges as a viable alternative given its proven antioxidant properties, established production infrastructure, and superior cost-effectiveness.

As a potent water-soluble antioxidant, vitamin C directly participates in redox reactions with reactive oxygen species (ROS), neutralizing them into non-harmful compounds and halting free radical-initiated chain oxidation processes. Furthermore, vitamin C regenerates other antioxidants—including vitamin E and glutathione peroxidase—thereby extending their functional periods and establishing a synergistic antioxidant network (12). Extensive previous animal studies have shown that vitamin C exerts beneficial effects on enhancing host immunity and improving production performance (13–15). For example, Hu et al. (16) reported that dietary inclusion of 300 mg/kg vitamin C in breeding geese markedly improved intestinal nutrient absorption and promoted yolk vitamin C deposition. In growing-finishing pigs, combined supplementation with vitamin C and niacinamide has been verified to reshape gut microbiota, mitigate intestinal inflammatory responses, and enhance meat redness (17). Nevertheless, the majority of such studies have focused on poultry and monogastric animals, whereas relevant studies regarding vitamin C in dairy cows are still limited. Because dairy cows were historically believed to meet their vitamin C requirements through hepatic biosynthesis, dietary supplementation has received insufficient attention in dairy management (18). However, recent evidence indicates that heat exposure, transport stress, or hepatic dysfunction reduces vitamin C concentrations in dairy cattle, likely due to both impaired endogenous synthesis and heightened metabolic demands (19). The molecular structure of vitamin C renders it highly susceptible to oxidative degradation when exposed to atmospheric oxygen, a process accelerated by feed processing conditions (e.g., elevated temperatures, UV exposure, metallic catalysts). Additionally, ruminal microbial metabolism further compromises dietary vitamin C bioavailability in ruminants compared to monogastric species (20). These factors predispose heat-stressed dairy cows to vitamin C deficiency. We therefore hypothesize that supplemental vitamin C-2-phosphate administration during thermal stress could bolster antioxidant defenses in both ruminal microbiota and the host organism, ultimately improving productivity. To test this hypothesis, this study systematically evaluates vitamin C-2-phosphate supplementation effects on key physiological parameters—respiratory rate, rectal temperature, production metrics, ruminal fermentation profiles, and circulating antioxidant markers—in heat-stressed dairy cows. These investigations establish the groundwork for mechanistic explorations into vitamin C’s regulatory pathways.

## 2 Materials and methods

This experiment was conducted at a commercial dairy farm in Hohhot City, Inner Mongolia Autonomous Region, China. All animal experimental protocols involved in this study were approved by the Animal Ethics Committee of Inner Mongolia Agricultural University, with the approval number [2019040].

### 2.1 Animals, experimental design, and diets

Thirty multiparous Holstein cows with a body weight of 659.6 ± 18.4 kg, a parity of 2.83 ± 0.65, an average lactation day of 93.3 ± 9.3 days, and a daily milk yield of 44 ± 4.3 kg were selected. These cows were divided into three treatment groups via a randomized complete block design based on their daily milk yield, lactation days, and body weight, with 10 cows in each group. The control group (CON) was fed a basal diet, while treatment group 1 (LVC) received the basal diet supplemented with 20 g/head/d of Vitamin C-2-phosphate, and treatment group 2 (HVC) was offered the basal diet supplemented with 40 g/head/d of Vitamin C-2-phosphate. The basal diet in this experiment was a total mixed ration (TMR), and its ingredient composition and nutrient levels are presented in Table 1. Vitamin C-2-phosphate was provided by Heilongjiang NHU Biotech Co., Ltd. (Heilongjiang, China), with an effective vitamin C content of 35%. During the experiment, all dairy cows had ad libitum access to fresh water and feed. The cows were fed three times daily at 06:30, 12:30, and 18:30, and milking was performed three times daily at 06:00, 12:00, and 18:00. This experiment was conducted during the summer months (July–September). The total experimental period lasted for 6 weeks, including a 1-week adaptation period and a 5-week formal experimental period.

**Table 1:** Composition and nutrient levels of the basal diet (dry matter basis %).

Items	Content
Ingredients	
Corn silage	30.99
Corn grain flaked	13.44
Corn grain ground fine	10.08
Alfalfa hay	10.83
Cottonseed	4.34
Wet brewers’ grain	4.29
Cane molasses	0.88
Sodium bicarbonate	1.07
Bergafat	0.80
Soybean meal	12.77
Rapeseed meal	3.73
Extruded soybean	1.01
Corn gluten meal	1.03
Premix^1^	4.74
Nutritional level	
DM	49.38
CP	17.58
EE	5.62
NDF	25.75
ADF	16.31
Crude ash	7.08
Ca	0.69
P	0.43
NEL^2^, Mcal/kg DM	1.72

^1^Each kg of the premix contains 100,000 IU vitamin A, 30,000 IU vitamin D₃, 1,600 IU vitamin E, 280 mg copper (Cu), 1,100 mg manganese (Mn), 2,400 mg zinc (Zn), and 200 mg cobalt (Co).

^2^NEL values were estimated according to the NRC (2001) system.

### 2.2 Temperature and humidity during the experiment

A temperature-humidity recorder (Xuzhou Fala Electronic Technology Co., Ltd., Xuzhou, China) was placed at a height of 1.5 m above the ground in the middle of the experimental cowshed. This instrument recorded the ambient temperature and relative humidity every 5 min. The daily average THI was calculated using the 24-h average temperature and humidity data. The calculation formula for THI is as follows:


THI=(1.8×T+32)−[0.55−(0.0055×RH)]×(1.8×T−26)

Among the variables in the formula, T denotes ambient temperature, and RH denotes relative humidity.

### 2.3 Rectal temperature and respiratory rate

During the experiment, the rectal temperature (RT) and respiratory rate (RR) of each cow were measured once a week at 07:00, 13:00, and 19:00, respectively. Rectal temperature was measured using a veterinary electronic thermometer. The thermometer was inserted into the cow’s rectum and left in place for approximately 2 min; data were recorded once the reading stabilized. Respiratory rate was defined as the number of breaths per minute (breaths/min) of the cows. To minimize measurement error, each cow was observed continuously for 2 min to count the number of abdominal movements while the cow was in a recumbent position. The average number of breaths per minute was then calculated based on this 2-min observation.

### 2.4 Dry matter intake, milk yield, and milk composition

Samples of the total mixed ration (TMR) and orts (remaining feed) were collected daily. These samples were dried in an oven at 55 °C for 48 h until a constant weight was achieved, and the dry matter (DM) content was recorded. The dried feed samples were then ground and passed through a 1-mm sieve. All samples collected during the final week of the experiment were mixed for subsequent chemical composition analysis. The contents of dry matter (DM), crude protein (CP), ether extract (EE), and crude ash (Ash) were analyzed following the methods of the Association of Official Analytical Chemists (AOAC). The concentrations of neutral detergent fiber (NDF) and acid detergent fiber (ADF) were determined using a Fiber Analyzer (2000i, Ankom Technology, New York, USA).

During the experiment, daily milk yield was recorded using the GEA milking platform. On days 1, 17, and 35 of the experiment, 100 mL of 24-h composite milk samples were collected from each cow. The morning, midday, and evening milk samples were mixed at a ratio of 4:3:3, and potassium dichromate was added to the mixed samples as a preservative. Subsequently, milk fat, milk protein, lactose, somatic cell count (SCC), and milk urea nitrogen (MUN) were determined using FOSS Milk OscanTM FT + automatic milk analyzer (FOSS, Hillerod, Denmark). The calculation formula for energy-corrected milk (ECM) was consistent with that reported by Jiang et al. (21), while the calculation of 4% fat-corrected milk (FCM) adopted the method described by Li et al. (22). The specific formulas are as follows:


$$\begin{array}{l} \mathrm{ECM}\;(\mathrm{kg}/d)=(0.3246\times \text{daily milk yield},\mathrm{kg})+\\ (13.86\times \text{daily milk}\;\mathrm{fat}\;\text{yield},\mathrm{kg})+\\ \left(7.04\times \text{daily milk protein yield},\mathrm{kg}\right) \end{array}$$

$$\begin{array}{l} 4\%\mathrm{FCM}\;(\mathrm{kg}/d)=\;(0.4\times \text{milk yield}\;(\mathrm{kg}/d))+\;\\ \left(15\times \text{milk}\;\mathrm{fat}\;\text{yield}\;(\mathrm{kg}/d)\right) \end{array}$$

### 2.5 Rumen fluid collection and analysis

On the 1st day after the completion of the feeding trial, 40 mL of rumen fluid was collected from the oral cavity of dairy cows using the gastric tube negative pressure method. The pH value of the rumen fluid was measured immediately. Subsequently, the rumen fluid was divided into two aliquots and stored frozen at −40 °C for the determination of volatile fatty acids (VFA) and ammonia nitrogen (NH₃-N). Among them, VFA was detected by gas chromatography–mass spectrometry (GC–MS), and NH₃-N was determined by the phenol-hypochlorite colorimetric method. The specific detection procedures referred to the method described by He et al. (23).

### 2.6 Blood collection and analysis

One hour before the morning feeding on the final day of the experiment, 15 mL of blood was collected from the caudal vein of each Holstein dairy cow. The blood samples were centrifuged at 2000 × g for 15 min to separate the serum, which was then stored at −40 °C for subsequent determination of blood indices. The serum indices, including glutathione peroxidase (GSH-P_X_), SOD, CAT, MDA, T-AOC, and HSP-70, were determined by enzyme-linked immunosorbent assay (ELISA). This analysis was commissioned by Yingjie (Nanjing) Intelligent Technology Co., Ltd. (Nanjing, Jiangsu, China).

### 2.7 Economic benefit analysis

Based on the average daily dry matter intake of dairy cows, milk yield, and the selling price of fresh milk, feed costs and milk production revenue were calculated systematically, ultimately deriving the overall economic benefit. The calculation formulas followed the methodologies documented in Reference (24).

Milk production revenue [CNY/(cow·day)] = Average daily milk yield × Fresh milk price per kilogram.

Feed cost [CNY/(cow·day)] = Average daily dry matter intake × TMR dry matter price per kilogram.

Net economic benefit [CNY/(cow·day)] = Milk output revenue − feed input cost.

### 2.8 Statistical analysis

Before analysis, the Shapiro–Wilk and Levene tests were used to examine the normality and homogeneity of variance of the data, and all data met the assumptions of normality and equal variance. Subsequently, all data were subjected to one-way analysis of variance (ANOVA) using SPSS statistical software (Version 23.0 for Windows; SPSS, Chicago, USA). The Tukey post-hoc test was applied to analyze differences between the two treatment groups. Data are presented as means and standard errors, with *p* < 0.05 considered statistically significant.

## 3 Results

### 3.1 Monitoring of THI during the experiment

**Figure 1:**
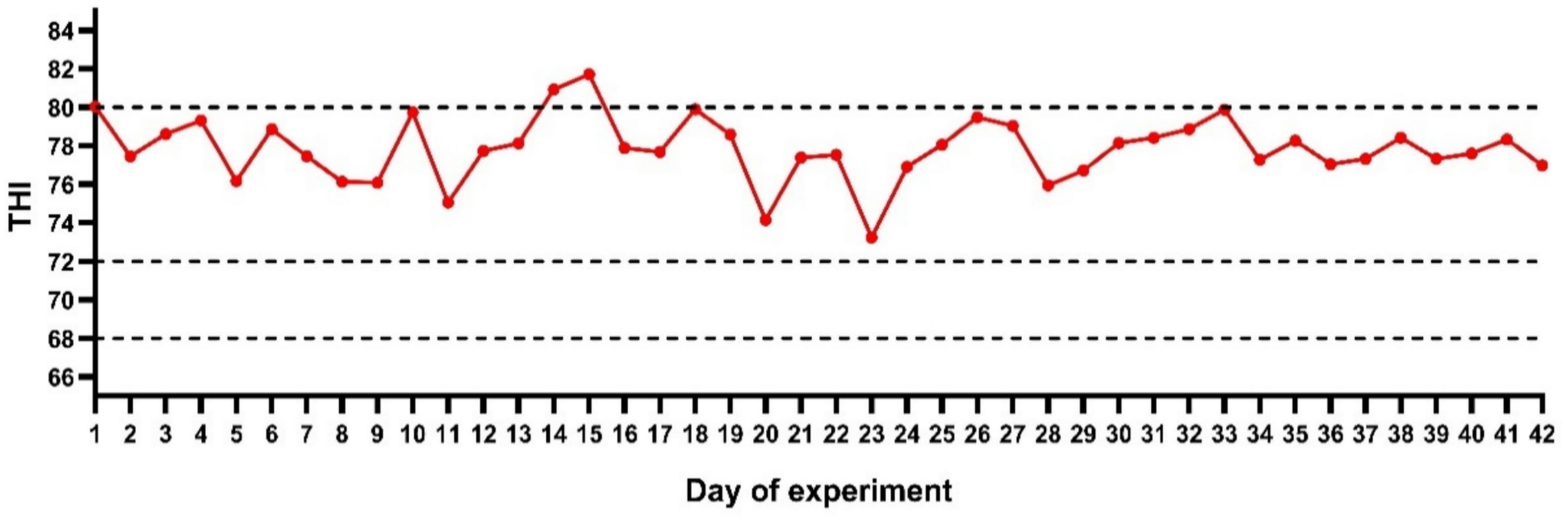
Changes in the THI during the experimental period.

As shown in Figure 1, the minimum THI during the experiment was 73.24, the maximum THI was 81.72, and the average THI was 77.86. Throughout the entire experimental period, the THI remained above 72, indicating that the dairy cows were in a state of moderate heat stress during the experiment, and reached severe heat stress during certain periods.

### 3.2 Effects of vitamin C supplementation on rectal temperature and respiratory rate

As shown in Table 2, dietary supplementation with vitamin C had no significant effect on the RR or RT of dairy cows during heat stress.

**Table 2:** Effects of vitamin C supplementation on respiratory rate and rectal temperature in heat-stressed dairy cows.

Items	Treatment			SEM	*p*-value	
	CON	LVC	HVC		Treatment	Linear
Respiratory rate (breaths/ min)						
07:00	54.30	52.90	52.60	0.58	0.46	0.25
13:00	74.20	72.60	71.90	1.10	0.70	0.41
19:00	59.80	58.60	59.20	0.50	0.64	0.64
Average	62.77	61.37	61.23	0.42	0.27	0.14
Rectal temperature (°C)						
07:00	38.49	38.37	38.38	0.03	0.29	0.20
13:00	39.13	39.03	38.94	0.06	0.41	0.19
19:00	38.68	38.52	38.52	0.04	0.11	0.07
Average	38.77	38.64	38.62	0.03	0.14	0.07

### 3.3 Effects of vitamin C supplementation on lactation performance of dairy cows

Table 3 presents the effects of vitamin C supplementation on DMI, milk yield, and milk composition in heat-stressed dairy cows. With the increase in dietary vitamin C supplementation level, the ECM yield, milk protein concentration, and milk protein yield significantly increased (*p* < 0.05), while the SCC significantly decreased (*p* < 0.05).

**Table 3:** Effects of vitamin C supplementation on lactation performance in heat-stressed dairy cows.

Items	Treatments			SEM	*p*-value	
	CON	LVC	HVC		Treatment	Linear
Dry matter intake (DMI, kg/d)	26.55	26.69	26.74	0.10	0.71	0.42
Daily milk yield (kg/d)	43.19	43.55	43.90	0.15	0.17	0.06
Feed conversion ratio (FCR)	1.63	1.63	1.65	0.01	0.77	0.50
4% FCM (kg/d)	41.28	41.71	42.16	0.20	0.21	0.08
ECM (kg/d)	45.66^b^	46.27^ab^	46.87^a^	0.22	0.08	0.03
Milk fat (%)	3.71	3.72	3.74	0.02	0.84	0.56
Milk protein (%)	3.11^b^	3.16^ab^	3.20^a^	0.01	0.02	<0.01
Lactose (%)	5.17	5.17	5.16	0.01	0.83	0.56
SCC, ×10^3^/mL	179.9^b^	173^ab^	161.5^a^	3.38	0.08	0.03
Milk urea nitrogen (MUN, mg/dL)	14.27	13.81	13.32	0.20	0.17	0.06
Milk fat yield (kg/d)	1.60	1.62	1.64	0.01	0.34	0.15
Milk protein yield (kg/d)	1.35^b^	1.38^ab^	1.40^a^	0.01	0.01	0.01
Lactose yield (kg/d)	2.24	2.25	2.27	0.01	0.36	0.16

^a-b^Values within the same row that are marked with different lowercase letters are significantly different from each other (*p* < 0.05).

### 3.4 Effects of vitamin C supplementation on rumen fermentation of dairy cows

Table 4 mainly illustrates the effects of vitamin C supplementation on rumen fermentation in heat-stressed dairy cows. With the dietary supplementation of vitamin C, the total volatile fatty acid (TVFA) concentration and acetate concentration significantly increased (*p* < 0.05). Additionally, the NH₃-N concentration in the LVC and HVC groups was significantly lower than that in the CON group (*p* < 0.05).

**Table 4:** Effects of vitamin C supplementation on rumen fermentation in heat-stressed dairy cows.

Items	Treatments			SEM	*p*-value	
	CON	LVC	HVC		Treatment	Linear
Rumen pH	6.33	6.39	6.38	0.02	0.36	0.25
TVFA, mM	99.44^b^	102.21^ab^	103.87^a^	1.04	0.05	0.02
Acetate (A), mM	60.14^b^	62.98^a^	64.13^a^	0.63	0.01	<0.01
Propionate (P), mM	23.73	22.83	23.00	0.22	0.89	0.65
Butyrate, mM	12.44	12.33	12.65	0.25	0.88	0.75
Isobutyrate, mM	1.1	1.15	1.06	0.03	0.6	0.60
Valerate, mM	1.49	1.47	1.49	0.03	0.95	0.98
Isovalerate, mM	1.53	1.46	1.54	0.04	0.72	0.94
A: P ratio	2.65	2.76	2.79	0.04	0.22	0.11
NH₃-N, mg/dL	13.14^a^	11.60^b^	10.70^b^	0.35	0.01	<0.01

^a-b^Values within the same row that are marked with different lowercase letters are significantly different from each other (*p* < 0.05).

### 3.5 Effects of vitamin C supplementation on blood indices of dairy cows

**Figure 2:**
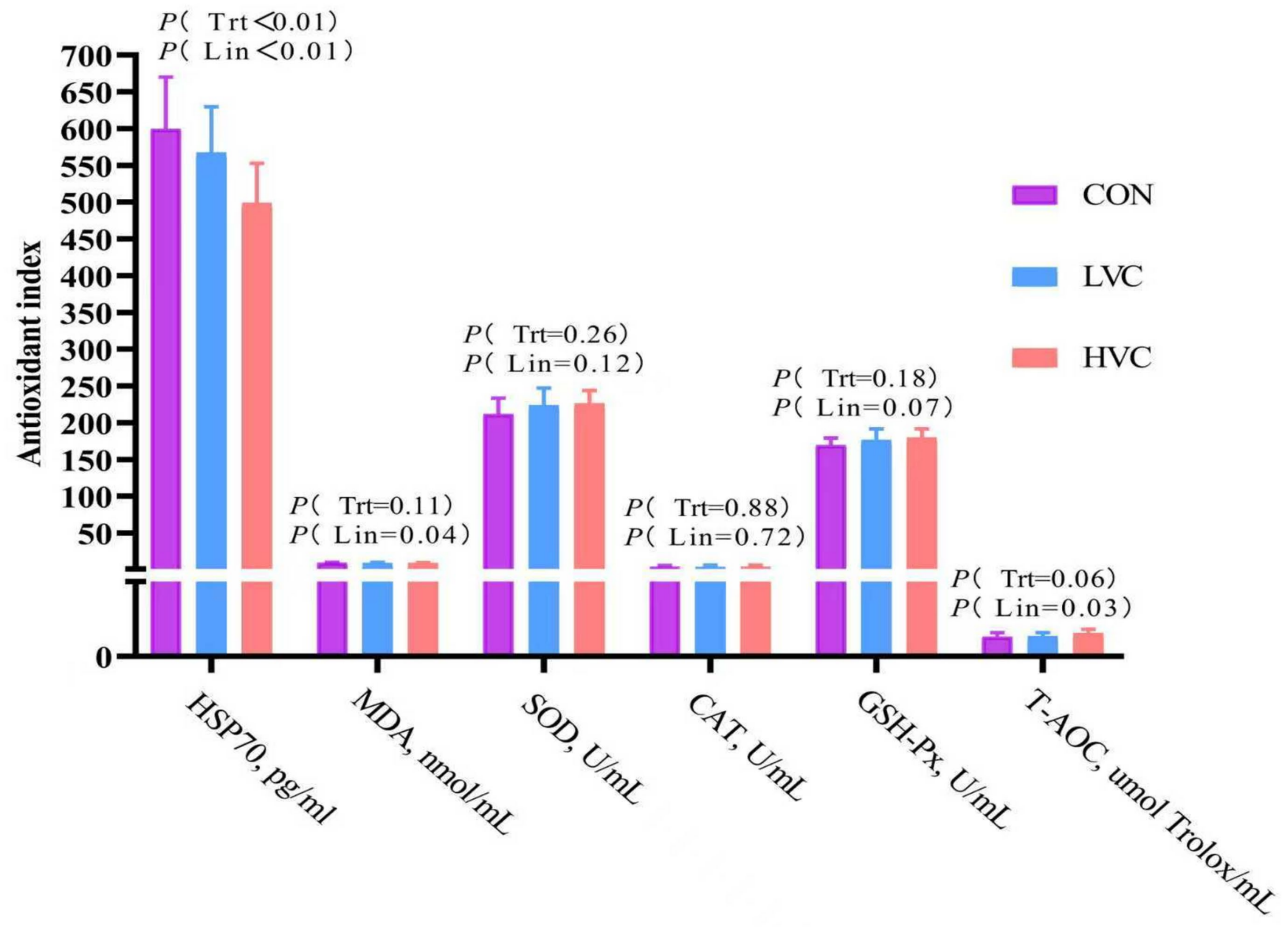
Effects of vitamin C supplementation on antioxidant status in heat-stressed dairy cows.

Figure 2 mainly presents the effects of vitamin C supplementation on blood antioxidant indices and heat stress factors in heat-stressed dairy cows. The T-AOC content significantly increased with vitamin C supplementation (*p* < 0.05). In contrast, the levels of HSP70 and MDA significantly decreased with vitamin C supplementation (*p* < 0.05).

### 3.6 Effects of vitamin C supplementation on the economic benefits of dairy cows

The economic benefits of dietary vitamin C supplementation for dairy cows are presented in Table 5. The milk price was set at 3 CNY/kg during the calculation period. The data showed that the diet cost was 78.71 CNY/(d·head) in the CON, and increased to 79.67 CNY/(d·head) and 80.36 CNY/(d·head) in the LVC and HVC groups, respectively. Meanwhile, milk production revenue rose from 129.56 CNY/(d·head) in the CON group to 130.65 CNY/(d·head) and 131.71 CNY/(d·head) in the LVC and HVC groups, respectively. Overall, the economic benefits were improved by 0.13 CNY/(d·head) and 0.5 CNY/(d·head) in the LVC and HVC groups compared with the CON group, respectively.

**Table 5:** Effects of vitamin C supplementation on the economic benefits of dairy cows.

Items	Treatments		
	CON	LVC	HVC
Feed intake DMI (kg/d)	26.55	26.69	26.74
Milk price (CNY/kg)	3.00	3.00	3.00
Diet cost per kilogram of dry matter [CNY/kg DM]	2.96	2.99	3.01
Milk yield (kg/d)	43.19	43.55	43.90
Milk production benefit [CNY/(cow·day)]	129.56	130.65	131.71
Diet cost [CNY/(cow·day)]	78.71	79.67	80.36
Diet cost per kilogram of milk (CNY/kg)	1.82	1.83	1.83
Economic benefit [CNY/(cow·day)]	50.85	50.98	51.35

## 4 Discussion

### 4.1 Effects of vitamin C supplementation on rectal temperature and respiratory rate

Selective breeding for enhanced productivity has progressively lowered the heat stress threshold in high-yield dairy cows, which exhibit thermal discomfort at a daily mean THI > 68 and severe production declines when THI exceeds 75 (8). During the experiment, the THI ranged from 73.24 to 81.72, indicating that the experimental cows were exposed to distinct heat stress, which was further verified by the RR and RT data. Under normal physiological conditions, the rectal temperature (RT) and respiratory rate (RR) of dairy cows generally do not exceed 38.5 °C and 60 breaths/min, respectively. However, heat stress triggers immediate elevations in both parameters to promote convective and evaporative heat dissipation (25). Some studies have found that ascorbic acid supplementation effectively decreases the rectal temperature of goats (26). However, no beneficial effects of ascorbic acid on body temperature and respiratory rate were observed in the present study. The discrepancy may be attributed to differences in the rearing environment. Similarly, no beneficial effects of vitamin C on respiratory rate (RR) and rectal temperature (RT) were observed in ovine and rabbit heat stress models. Suggesting minimal therapeutic potential for vitamin C in directly mitigating acute thermoregulatory responses (27, 28). This non-significant impact likely arises from the heightened sensitivity and rapid responsiveness of thermoregulatory indicators relative to production variables like milk yield or feed intake, which exhibit delayed thermal stress responses. These findings imply that alternative interventions targeting cutaneous heat dissipation mechanisms may prove more efficacious for managing immediate thermal stress reactions in bovine systems.

### 4.2 Effects of vitamin C supplementation on lactation performance of dairy cows

Compared with its effects on RR and RT, farmers may place greater emphasis on the impacts of heat stress on production performance. It is well known that both milk yield and milk quality decrease in dairy cows during heat stress (44). Our experiment showed no significant improvement in dry matter intake (DMI) after vitamin C supplementation, which is consistent with the findings of Kim et al. (29) using rumen-protected vitamin C. This result may be attributed to the well-documented inverse relationship between rectal temperature and feed intake: elevated rectal temperature suppresses the hypothalamic feeding centers, thereby reducing appetite (30, 31). In the present study, DMI remained unchanged, whereas daily milk yield and fat-corrected milk tended to increase, and energy-corrected milk (ECM) was significantly improved. The pronounced ECM elevation primarily reflects increased milk protein yield, corroborating Semsirmboon et al.’s (32) observations in heat-stressed dairy goats receiving ascorbic acid. These improvements may be attributed to the capacity of ascorbic acid to neutralize reactive oxygen species (ROS) in mammary tissue, thereby alleviating endoplasmic reticulum stress induced by the accumulation of misfolded proteins. Such inhibition of the PERK/eIF2α pathway contributes to the restoration of protein synthesis machinery (33). Nevertheless, the lack of detection for relevant gene expression constitutes a deficiency of this trial, as well as a key direction for subsequent in-depth investigations. Concurrently, decreased ROS levels alleviate oxidative damage to mammary immune cells, thereby reducing milk somatic cell count (SCC), a critical indicator of udder health (5, 34). This mechanism also accounts for the reduction in SCC observed in the present study. By preserving phagocytic activity and suppressing inflammation, vitamin C enhances systemic immunity, potentially explaining the positive correlation between tissue ascorbic acid concentrations and milk productivity in high-yielding cows (35). Collectively, these findings underscore vitamin C’s multifaceted roles in counteracting heat stress pathologies beyond mere thermal regulation.

### 4.3 Effects of vitamin C supplementation on rumen fermentation of dairy cows

The rumen serves as a pivotal organ governing productivity in ruminants, with its health status directly correlating to dairy cow performance metrics. Prolonged heat stress induces ruminal acidosis through multiple pathways: altered feeding selectivity, respiratory saliva loss, and dysbiosis of resident microbiota (36, 37). These microbial communities facilitate nutrient digestion via enzymatic secretion, underscoring the importance of population stability for digestibility optimization (38). However, exogenous stressors compromise microbial membrane integrity, triggering protein denaturation and subsequent bacterial apoptosis (39). During bovine oxidative stress, systemic ROS infiltrate the rumen via hematogenous or secretory routes, impairing microbial metabolic activity. Our findings demonstrate that vitamin C supplementation elevated total volatile fatty acids and acetate concentrations—likely attributable to ROS scavenging that preserved acetogenic bacterial populations. Concurrent reductions in ammonia nitrogen and elevations in milk protein suggest enhanced microbial protein synthesis efficiency. Notably, despite TVFA accumulation and reduced ammonia nitrogen, stable ruminal pH was maintained, potentially due to diminished lactate and other organic acids. Prior research demonstrated heat stress-induced proliferation of lactic acid producers alongside suppression of acetogens (37), whereas our results indicate vitamin C reversed this trend through interspecies competition modulation. While vitamin C augmented fermentation intensity without altering fermentation profiles, direct microbial quantification remains an unaddressed experimental limitation. Future investigations incorporating metagenomic analyses will elucidate precise mechanisms underlying these observations.

### 4.4 Effects of vitamin C supplementation on blood indices of dairy cows

Superoxide dismutase acts as the primary antioxidant defense system component, converting superoxide radicals into hydrogen peroxide, which is subsequently detoxified to water by catalase and glutathione peroxidase (40). The phosphoinositide 3-kinase-protein kinase B (PI3K-Akt) pathway critically regulates antioxidant enzymes, including SOD and GSH-Px, to maintain redox homeostasis. Under heat stress conditions, ROS accumulation triggers significant PI3K-Akt pathway suppression, disrupting systemic redox balance (41). Prior studies confirm that heat stress reduces SOD and CAT activities while elevating malondialdehyde and ROS levels in bovine tissues, causing oxidative stress (5). Notably, this study did not measure critical stress-related indicators such as cortisol, triiodothyronine (T3) and thyroxine (T4), which represents a limitation of the present trial. These parameters will be analyzed in future research to further clarify the regulatory mechanism of vitamin C in stressed dairy cows. Our experimental intervention demonstrated that dietary vitamin C supplementation significantly decreased serum MDA and heat shock protein 70 concentrations, elevated total antioxidant capacity, and showed a trend toward increased GSH-Px activity. These findings align with Nwunuji et al.’s (42) observation of reduced MDA levels in transported goats receiving vitamin C. Given MDA’s role as a lipid peroxidation biomarker and T-AOC’s reflection of systemic antioxidant potential, our results validate vitamin C’s efficacy in enhancing antioxidant defenses. The observed GSH-Px activity modulation correlates with established mechanisms where vitamin C regenerates reduced glutathione from oxidized glutathione, providing essential substrate for GSH-Px synthesis (12). Heat stress-induced ROS upregulate heat shock proteins (HSPs), particularly HSP70—a sensitive oxidative stress indicator (43). Vitamin C supplementation’s reduction of circulating HSP70 provides further evidence of its ROS-scavenging properties. Together, these data demonstrate that vitamin C-2-phosphate achieves favorable bioavailability in dairy cattle, effectively enhancing whole-body antioxidant capacity through multiple complementary pathways.

## 5 Conclusion

The data of the present study demonstrated that high-dose vitamin C-2-phosphate exerted more positive effects on improving lactation performance, enhancing the body’s antioxidant status, and increasing economic benefits in dairy cows. This study can provide a theoretical basis for nutritional intervention strategies in dairy cows suffering from heat stress, parturition stress, and transportation stress. Currently, studies regarding the effects of vitamin C on dairy cows remain relatively scarce, and its underlying mechanisms of action in the gastrointestinal tract, mammary gland, and the whole body of dairy cows are still unclear. Therefore, further research is warranted to explore the effects of different forms of ascorbic acid and their optimal supplemental levels on enhancing the antioxidant capacity and immune function of dairy cows.

## Data Availability

The original contributions presented in the study are included in the article/supplementary material, further inquiries can be directed to the corresponding author.
